# Pulmonary lymphangioleiomyomatosis and renal angiomyolipoma in a patient with systemic lupus erythematosus: A case report

**DOI:** 10.1097/MD.0000000000030554

**Published:** 2022-09-23

**Authors:** Jeong Suk Koh, Sina Oh, Chaeuk Chung

**Affiliations:** a Division of Pulmonology and Critical Care Medicine, Department of Internal Medicine, College of Medicine, Chungnam National University, Daejeon, 301-721, Republic of Korea; b College of Medicine, Chungnam National University, Daejeon 35015, Republic of Korea; c Infection Control Convergence Research Center, Chungnam National University School of Medicine, Daejeon 35015, Republic of Korea.

**Keywords:** angiomyolipoma, lymphangioleiomyomatosis, systemic lupus erythematous, tuberous sclerosis complex

## Abstract

The co-incidence of systemic lupus erythematosus (SLE) and tuberous sclerosis with pulmonary lymphangioleiomyomatosis (LAM) and renal angiomyolipoma (AML) is rare. In such patients, the rupture of renal AML may result in fatal circumstances, but this may be preventable.

**Methods:**

A 22-year-old Asian woman with SLE was admitted to our hospital with severe left-flank pain. Imaging studies showed the bilateral rupture of multiple renal AMLs.

**Results:**

The patient underwent emergency selective transcatheter embolization (TE) of the left renal artery. After TE and massive hydration, the patient complained of dyspnea and postembolization syndrome with fever. The chest computed tomography (CT) revealed pulmonary LAM, pulmonary edema with bilateral pleural effusions, and pneumonic consolidation. After the emergency procedure, the patient was treated with intravenous administration of antibiotics, diuretics, and nonsteroidal anti-inflammatory drugs for 10 days. The patient recovered favorably and was discharged 20 days after the treatment. She was diagnosed with renal AML and pulmonary LAM along with facial angiofibromas as well as tuberous sclerosis complex (TSC), although she had no TSC1 or TSC2 gene mutations.

**Conclusion:**

Although rare, SLE may coexist with TSC, along with LAM and AML, with a risk of AML rupture. The activation of the mTOR signaling pathway is shared between SLE and TSC. Thus, in patients with SLE, clinicians should consider imaging studies, such as kidney sonography and chest CT, to screen for possible manifestation of AML and LAM.

## 1. Introduction

Systemic lupus erythematosus (SLE) is an autoimmune disease mediated by autoantibodies and immune complexes. Most of the affected patients (90%) were women of childbearing ages. The factors involved in SLE pathogenesis include genetic background, environmental influences, and epigenetics. Several studies have shown that the mammalian target of rapamycin (mTOR) pathway plays an important role in the pathogenesis of SLE. The mammalian target of rapamycin regulates the growth and metabolism of eukaryotic cells according to signals from growth factors and nutrients and is deeply involved in immune responses. Rapamycin has been shown to expand T regulatory cells, thereby suppressing the activation and proliferation of autoreactive T cells and reducing the activity of SLE.^[1,2]^

The mTOR pathway is also involved in the pathogenesis of tuberous sclerosis complex (TSC), which results from mutations in the TSC1 (encoding hamartin) or TSC2 (encoding tuberin) gene. Tuberous sclerosis complex is an autosomal dominant disease that invades multiple systems, with the most commonly affected organs being the brain, skin, kidneys, eyes, heart, and lungs.^[2]^ Thus, patients with SLE may be at risk of developing TSC, because both diseases involve the mTOR pathway. Tuberous sclerosis complex is also closely related to pulmonary lymphangioleiomyomatosis (LAM), a rare disease in women characterized by smooth muscle cell infiltration and cystic destruction of the lungs,^[3]^ and renal angiomyolipoma (AML), a mesenchymal tumor and the most common benign tumor of the kidney. Angiomyolipoma occurs in 30%–60% of patients with LAM and may develop many years before its actual diagnosis.^[4]^

## 2. Case presentation

**Figure 1.**
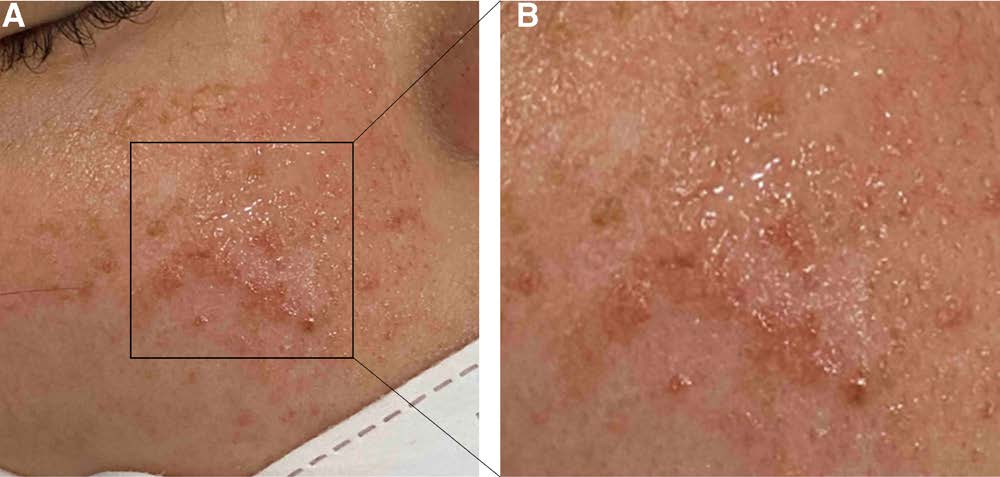
Facial fibroangiomas. (A) Multiple papules of various sizes distributed on the face, near the nose, and increasing in size. (B) Magnified view of the boxed lesion in (a).

The patient was a 22-year-old Asian woman, and her occupation was student. At the age of 15, she was diagnosed with SLE based on cutaneous manifestations (malar rash), a non-scarring alopecia, Raynaud’s phenomenon, thrombocytopenia, hypocomplementemia, and serological evidence (antinuclear antibody titer, 1:1280 [homogeneous pattern]; anti-dsDNA antibody level, 445.96 IU/mL; and anti-Sm antibody-positive) since 2014. She was treated with prednisolone (2.5 mg/day), hydroxychloroquine (200 mg/day), and methotrexate (4 times/week) with no complications. During treatment, her renal function was normal and she had no serious manifestations of SLE or significant laboratory abnormalities. However, she recently developed angiofibromas (Fig. 1) on her face near her nose, which increased in size.

**Figure 2.**
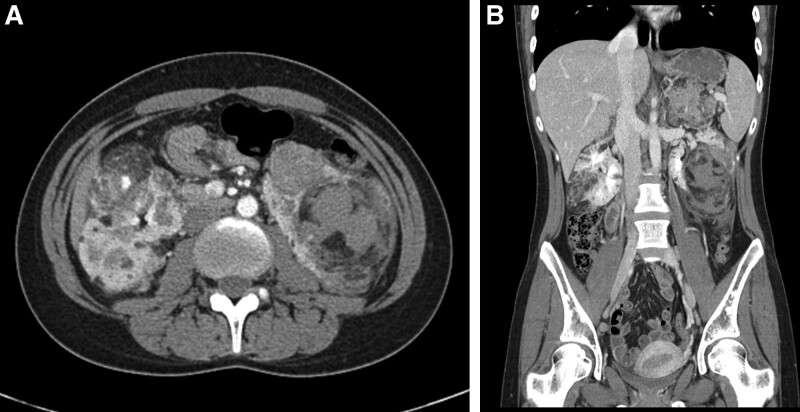
Abdominal computed tomography scan. (A) Axial CT image showing bilateral renal AMLs rupture with an intra-hemorrhagic component. (B) Coronal CT scan demonstrating AML rupture in the left kidney. CT = computed tomography, AML = angiomyolipoma.

**Figure 3.**
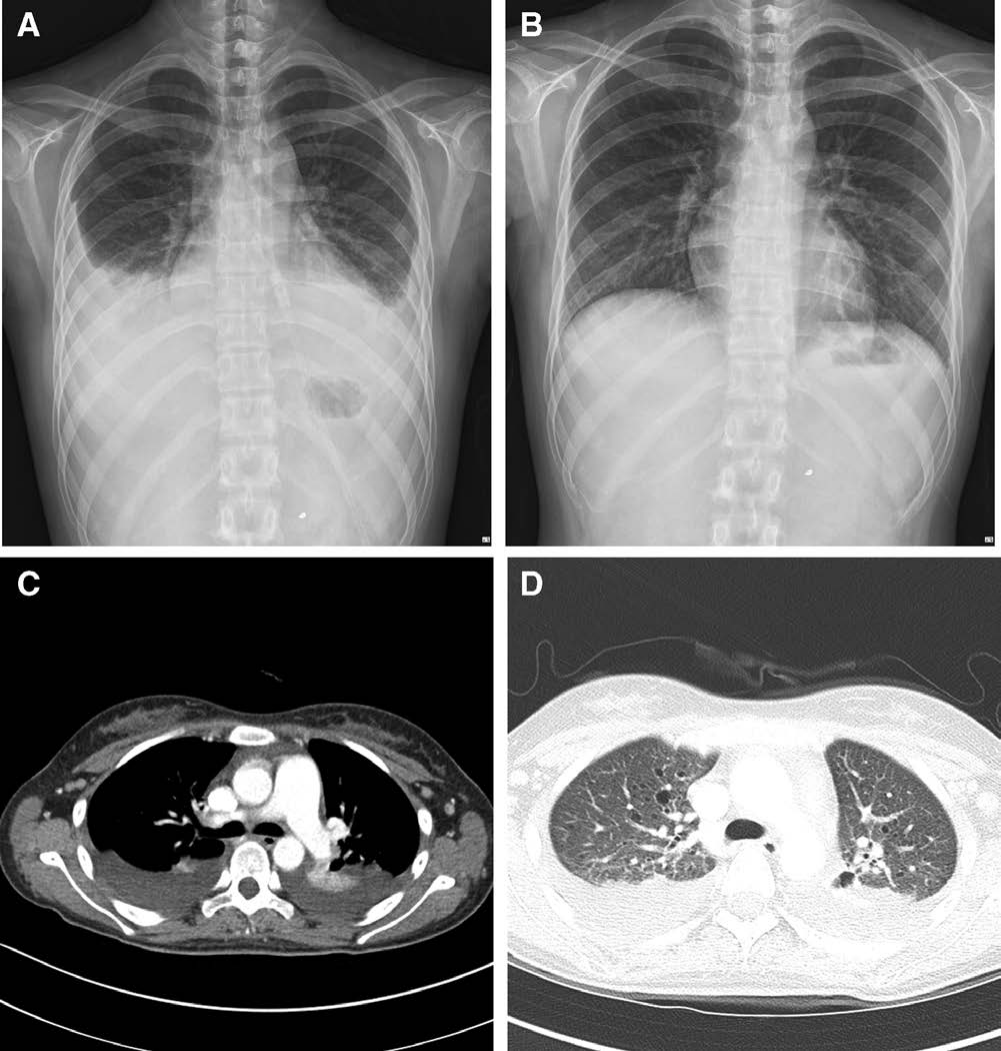
Chest images. (A) Chest X-ray showing bilateral pleural effusion after left renal selective TE. (B) Following treatment with diuretics and antibiotics, the patient’s chest X-ray findings markedly improved. (C) The mediastinal setting of the chest CT shows pleural effusion in both lungs. (D) The lung setting of the chest CT demonstrates multiple cystic lesions of the lung parenchyma. TE = transcatheter embolization, CT = computed tomography.

In October 2021, the patient presented with a fever and severe left flank pain. Her blood pressure was 106/65 mmHg, pulse rate was 118 bpm, respiratory rate 20/min, and body temperature 39.5°C. Imaging revealed multiple bilateral renal AMLs with an intrahemorrhagic component (Fig. 2). The patient underwent left renal selective transcatheter embolization (TE) for left AMLs rupture using PVA particle (contour^TM^, Boston Scientific) and microcoils (2mm, Nester). After TE and hydration, she presented with modified Medical Research Council (mMRC) grade 4 dyspnea and a persistent fever of 39°C. Chest computed tomography (CT) revealed pulmonary edema with bilateral pleural effusion, pneumonic consolidation, and pulmonary LAM (Fig. 3). Examination of the pleural effusion showed 4.0 g of protein/L (corresponding serum proteins:6.4 g/L), a lactate dehydrogenase (LDH) level of 1526 U/L (corresponding serum LDH:1543 U/L), and a glucose level of 98 mmol/L. She was treated with intravenous antibiotics (piperacillin/clavulanate, Chong Kun Dang Pharmaceutical Corp.), loop diuretics (furosemide, Daihan Pharm Co., Ltd.), and non-steroidal anti-inflammatory drugs (Nimesulide, JW Pharmaceutical). Pulmonary edema and pleural effusion resolved, and the patient was discharged after 20 days of treatment (Fig. 3). Although she presented with renal AML, pulmonary LAM, and facial angiofibromas, 3 of the major criteria for a definitive diagnosis of TSC (Table 1),^[5]^ she had no history of seizures or other neurological manifestations, and no TSC1 or TSC2 mutations were found. In addition, no TSC mutations were found in her family members, all without a history of SLE.

**Table 1:** Diagnostic criteria for tuberous sclerosis complex.

	
Major criteria	Hypomelanotic macules (≥3; at least 5 mm in diameter)
	Angiofibroma (≥3) or fibrous cephalic plaque
	Ungual fibromas (≥2)
	Shagreen patch
	Multiple retinal hamartomas
	Multiple cortical tubers and/or radial migration lines
	Subependymal nodule (≥2)
	Subependymal giant cell astrocytoma
	Cardiac rhabdomyoma
	Lymphangioleiomyomatosis*
	Angiomyolipomas (≥2)*
Minor criteria	“Confetti” skin lesions
	Dental enamel pits (≥3)
	Intraoral fibromas (≥2)
	Retinal achromic patch
	Multiple renal cysts
	Nonrenal hamartomas
	Sclerotic bone lesions

Definitive diagnosis of TSC: 2 major criteria or 1 major and 2 minor criteria.

Possible diagnosis of TSC: 1 major criterion or 2 or more minor criteria.

Genetic diagnosis: A pathogenic variant in *TSC1* or TSC2 is diagnostic for TSC (most TSC-causing variants are sequence variants that clearly prevent TSC1 or TSC2 protein production. Some variants compatible with protein production (e.g., some missense changes) are well established as disease-causing; other variant types should be considered with caution).

* A combination of the 2 major clinical features lymphangioleiomyomatosis and angiomyolipomas without other features does not meet the criteria for a definite diagnosis.

TSC = tuberous sclerosis complex.

## 3. Discussion

The frequency of TSC is between 1/6000 and 1/10,000 live births,^[6]^ and the simultaneous development of SLE and TSC is presumably even lower.

Our patient was diagnosed with SLE and clinical signs of TSC were detected 7 years later. A literature search revealed one case in which TSC was diagnosed before SLE developed and one case of an SLE patient who later developed TSC clinical symptoms, similar to our patient.^[7,8]^ TSC and SLE share an mTOR pathway in their pathophysiologies.

The patient visited our hospital with sudden flank pain. Abdominal CT revealed spontaneous AML rupture with multiple invasions in both kidneys. Therefore, arterial embolization was performed. The patient had no respiratory symptoms at the time of initial diagnosis of SLE. Clinically, patients with LAM can have a variety of symptoms including cough, shortness of breath, fatigue, and/or hemoptysis. Disease progression may be accompanied by effusion, lung collapse, or decreased lung function. After TE and massive hydration, the patient complained of dyspnea and exhibited a newly developed bilateral pleural effusion. As the effusion clearly improved after treatment with diuretics and antibiotics, it may not have been caused by LAM. In the present case, if TSC and AML were diagnosed in advance, renal AML rupture might have been preventable. In rare cases, SLE and TSC can occur simultaneously, and LAM and AML screening should be performed when treating SLE patients.

T cells in SLE are associated with disease manifestation and the mTOR pathway is involved in T cell differentiation and function. Rapamycin inhibits the mTOR pathway by increasing both the number of regulatory T cells and the production of IL-2, a cytokine essential for regulatory T cells.^[1]^ Activation of mTOR is thought to be a major cause of TSC and is associated with mutations in TSC1 and TSC2, thus implying a relationship between SLE and TSC. Therefore, it is recommended that *TSC* gene testing be conducted in patients with SLE who show any clinical manifestations that meet the diagnostic criteria for TSC. In our patient, although a *TSC* gene mutation was not identified, the possibility of TSC development could not be completely ruled out, and follow-up, such as an imaging examination, may be required.

**Figure 4.**
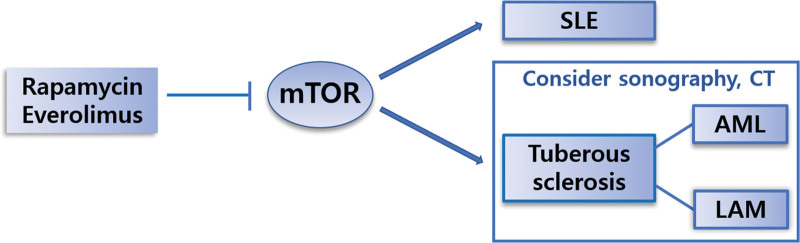
Schematic diagram of mammalian target of rapamycin signaling in systemic lupus erythematous and tuberous sclerosis complex with angiomyolipoma, and lymphangioleiomyomatosis. Both SLE and TSC rely on mTOR signaling. In rare cases, these two diseases can occur simultaneously. Therefore, it is recommended to consider LAM and AML screening by kidney sonography and chest CT scan when treating patients with SLE. SLE = systemic lupus erythematous, TSC = tuberous sclerosis complex, mTOR = mammalian target of rapamycin, LAM = lymphangioleiomyomatosis, AML = angiomyolipoma, CT = computed tomography.

Inhibition of the mTOR pathway reduces SLE activity, and the efficacy and safety of mTOR inhibitors in SLE clinical trials have been initially confirmed.^[9]^ The allosteric mTOR inhibitor everolimus, approved for the treatment of subependymal giant cell astrocytomas and renal AML in patients with TSC,^[10]^ may be beneficial in patients diagnosed with both SLE and TSC (Fig. 4).

In conclusion, this report describes the coexistence of TSC with LAM and renal AML rupture in a patient with SLE. Both SLE and TSC rely on mTOR signaling, which may account for their coexistence. Although such cases are rare, clinicians should consider imaging tests to screen for LAM and AML when treating SLE patients.

## Author contributions

JSK and SO wrote the manuscript. CC revised the manuscript and provided guidance. All the authors have read and approved the final manuscript.

Investigation: Jeong Suk Koh, Sina Oh, Chaeuk Chung.

Supervision: Chaeuk Chung.

Writing-original draft: Jeong Suk Koh, Sina Oh.

Writing-review & editing: Chaeuk Chung.
